# Cost-effectiveness analysis of parenteral iron therapy compared to oral iron supplements in managing iron deficiency anemia among pregnant women

**DOI:** 10.1186/s13561-023-00474-3

**Published:** 2024-01-02

**Authors:** Somen Saha, Devang Raval, Komal Shah, Deepak Saxena

**Affiliations:** Indian Institute of Public Health, Gandhinagar, Gujarat India

**Keywords:** Maternal anemia, Oral iron therapy, IV iron sucrose, Cost-effectiveness, Health technology assessment, India

## Abstract

**Objective:**

This study compared the clinical efficacy and cost-effectiveness of parenteral iron, using intravenous iron sucrose (IVIS) therapy against the standard regimen of oral iron (OI) therapy for managing iron-deficiency anemia (IDA) among pregnant women in a natural primary care setting in Gujarat.

**Design:**

A prospective cost-effectiveness study was conducted in natural programme setting wherein 188 pregnant women in their 14 to 18 weeks with moderate and severe anemia women enrolled from two districts of Gujarat, and 142 were followed up until the post-partum phase. The intervention group comprised of 82 participants who were administered IVIS, while the comparison group comprised of 106 participants who were put on OI therapy. Hemoglobin (Hb) levels were measured at periodic intervals, first during enrollment and then during each month of pregnancy period and finally on the 42nd day of the post-natal period.

**Outcome measures:**

Change in mean Hb level from baseline was the primary outcome, while the incidence of morbidity and mortality was a secondary outcome measure.

**Results:**

The intervention group showed a significant incremental mean change in Hb level from 8.2 g/dl to 11.45 g/dl at the fourth follow-up, while the control group's mean Hb level reduced from 9.99 g/dl to 9.55 g/dl. The discounted cost per beneficiary for IVIS was US$ 87, while that for OI was US$ 49. The incremental cost-effectiveness ratio (ICER) was US$ 9.84, which is 0.049% of India's per capita GDP.

**Conclusion:**

IVIS therapy was more clinically effective and cost-effective than OI therapy among pregnant women for management of moderate and severe anemia.

## Introduction

Maternal anemia is a major public health concern in India, with a prevalence rate of 52.2% among pregnant women aged 15–49 years, as indicated by NFHS 05 data (HB levels < 11.0 g/dl) [1]. The association of anemia among pregnant women with adverse birth outcomes such as neonatal deaths, premature deliveries, and low birth weights, pregnancy induce hypertension and pre-eclampsia are well documented [2, 3] it will also increase the healthcare costs for the management of these conditions. Oral iron tablets are the primary protocol for first-line treatment and management of anemia. Still, compliance with this mode of treatment is minimal due to several adverse side effects including vomiting, epigastric discomfort, and impaired absorption [4]. Furthermore, oral iron is ineffective for treating moderate to severe anemia detected during the late stages of pregnancy [5]. Administering intravenous iron sucrose (IVIS) has made parenteral therapy a viable option for pregnant women as it has proven to be an effective alternative to oral treatment [6]. In addition to its rapid absorption, the intravenous (IV) method is recognized for its reduced occurrence of hypersensitivity reactions (7). In recent times Ferric carboxy maltose (FCM) has been the most effective treatment option for the management of anemia among pregnant and lactating women [8, 9]. Several randomized controlled trials [10–12] have shown positive outcome with parenteral therapy using IVIS or FCM. However, under programmatic conditions there are dearth of evidence that compared the cost-effectiveness of parenteral therapy with OI for the improvement of hemoglobin levels. Unfortunately, in Indian contexts, no comprehensive cost analysis has been conducted (encompassing the expenses borne by the healthcare system) for the two management protocols of anemia among pregnant women [13]. A cost-effectiveness analysis comparing oral and injectable iron therapies was carried out in Uttar Pradesh in a primarily hospital-based setting, but without accounting for health system costs. Additionally, studies conducted by Jose et al. [5] and Mahey et al. [5] also neglected the inclusion of health system expenses in their cost-effectiveness evaluations.”

### Aim of the study

The study aimed to compare clinical efficacy and cost-effectiveness of the IVIS therapy with oral iron therapy among pregnant women with IDA in a programmatic setting at Banaskantha and Devbhoomi Dwarka district of Gujarat, India.

## Methodology

### Study population

During 2020–21, a prospective study was conducted in two districts of Gujarat, namely Banaskantha and Devbhoomi Dwarka, which were selected purposively. The detailed protocol for the study has been published in BMJ open [14]. All pregnant women who were registered in their 14–18 weeks of gestation in both districts were listed. Only pregnant women who were diagnosed with moderate to severe anemia were considered for inclusion in the study.

### Sampled Population

The study was conducted in natural programme setting wherein all pregnant women (14 weeks–18 weeks) with moderate and severe anemia who were registered at selected primary health centres of the Sabarkantha and Devbhoomi Dwarka District, Gujarat. The control arm was constituted by the pregnant women having moderate anemia who are exclusively on oral iron (OI) supplements as in severe anemia no IDA is being treated by IVIS and Blood transfusion. In a previous study change in mean Hb levels was calculated to determine the sample size [14]. After taking OI, the Hb level changed from 9.75 g/dl to 11.06 g/dl with a standard deviation of 0.72. In contrast, after administering IVIS, the Hb level changed from 9.18 g/dl to 11.24 g/dl, with a standard deviation of 0.82. The difference of 0.75 g/dl in the change in hemoglobin levels between the two groups was used to calculate the required sample size for the current study using the formula *n* = [DEFF*Np(1-p)]/ [(d^2^/Z^2^_1-α/2_*(N-1) + p*(1-p)]. Thus, considering an alpha error and power of 5% and 80% respectively, the sample size was estimated to be 26 per group. At an assumed loss to follow-up of 20%, the sample size was estimated to 32 per group. Hence, the study's total calculated sample size was 128, with 32 pregnant women from each arm in the two districts. We enrolled 188 pregnant women, and 142 were followed-up until the post-partum phase.

### Study protocol

The research team recorded information about their sociodemographic characteristics, past obstetric history, pre-intervention assessment (including height, weight, and Hb levels), and any history of treatments taken during the baseline data collection. The pregnant women were monitored for six weeks after delivery, and their Hb levels were assessed every month during the pregnancy period and post-delivery on the 42nd day. Hemoglobin estimation was done by a laboratory technician from the PHC during each follow-up visit using a digital hemoglobinometer [12]. To ensure the use of the pills, enrolled pregnant women were asked to carry empty packets and also enquires were made about their pill intake and stool colour during every follow-up visit.

### Inclusion and exclusion criteria

Pregnant women diagnosed with mild and moderate anemia were administered oral iron (OI), while those diagnosed with moderate and severe anemia were given parenteral therapy through IVIS. Participants who had undergone a transfusion of blood within the last 4 months or required one during any intervention stage were excluded from the study. Women who had hemoglobinopathy including any types of known red cell disorders, or suffering from any types of chronic infections such as hepatitis or HIV or a past occurrence of any allergic reaction were excluded from the study.

### Patient and public involvement

Neither the patients nor public were involved in planning or design of the study. Informed Consent: In this study human participation involved so written information consent was obtained from the study participants.

### Health outcomes

The study team assessed health-related quality of life (HRQoL) using the EQ-5D-5L (EuroQol-5 Dimension, 5 Levels). EQ-5D-5L is a validated tool used during baseline and during each follow-up. The EQ-5D-5L questionnaire has five dimensions that evaluate aspects of health-related quality of life (HRQoL) including aspects of difficulty in mobility, in taking self-care, in performing usual activities, any pain or discomfort, and episodes of anxiety and or depression. Each dimensions of the tool has five possible response levels: The first level indicates no problems, the second level indicates slight problems, the third level indicates moderate problems, the fourth level indicates severe problems, and the fifth level indicates extreme problems or discomfort. Additionally, the questionnaire features a visual analogue scale (VAS) on which ask each respondents to rate their self-perceived status of health using a graduated scale that ranges from 0 to 100. Higher the score the better is the HRQoL. VAS provides a more direct measure of the respondent's state of health. The descriptive system of EQ-5D-5L provides the health profile of the individual by converting the scores into an index representing a von Neumann-Morgenstern utility value of the current state of health [13]. Each unique health state is determined by the level of problem or discomfort reported on each of the dimensions of EQ-5D. To convert the reported health states into a weighted health state index, scores from the EQ-5D preference weights were applied. The preference weights were obtained from general population samples and calculated using the Crosswalk Index calculator [15]. These weights range from 0 (dead) to 1 (full health) on a scale. This study used the Thai population weights to convert them into EQ-5D index scores.

### Measuring the cost of care

The estimated cost of care per beneficiary is determined from a societal perspective. Financial records and field interviews were used to gather costs associated with various heads, including therapy expenses, consumables, healthcare resources, out-of-pocket expenditures, and lost wages etc. Therapy costs for OI and IVIS were gathered from government-approved rate contracts and from rates notified in case of local bulk procurement. Consumables data were collected from the facility, including materials, supplies, quantity used per test, and unit price. Administrative records were reviewed, while research costs were excluded. Travel and wage losses in case of referral or in case of follow-up visits were obtained from field records. Costs are reported in INR and USD, with 1 USD equal to 79.58 INR (1$ ~ 79.58 INR.

### Measuring the cost-effectiveness

**Fig. 1:**
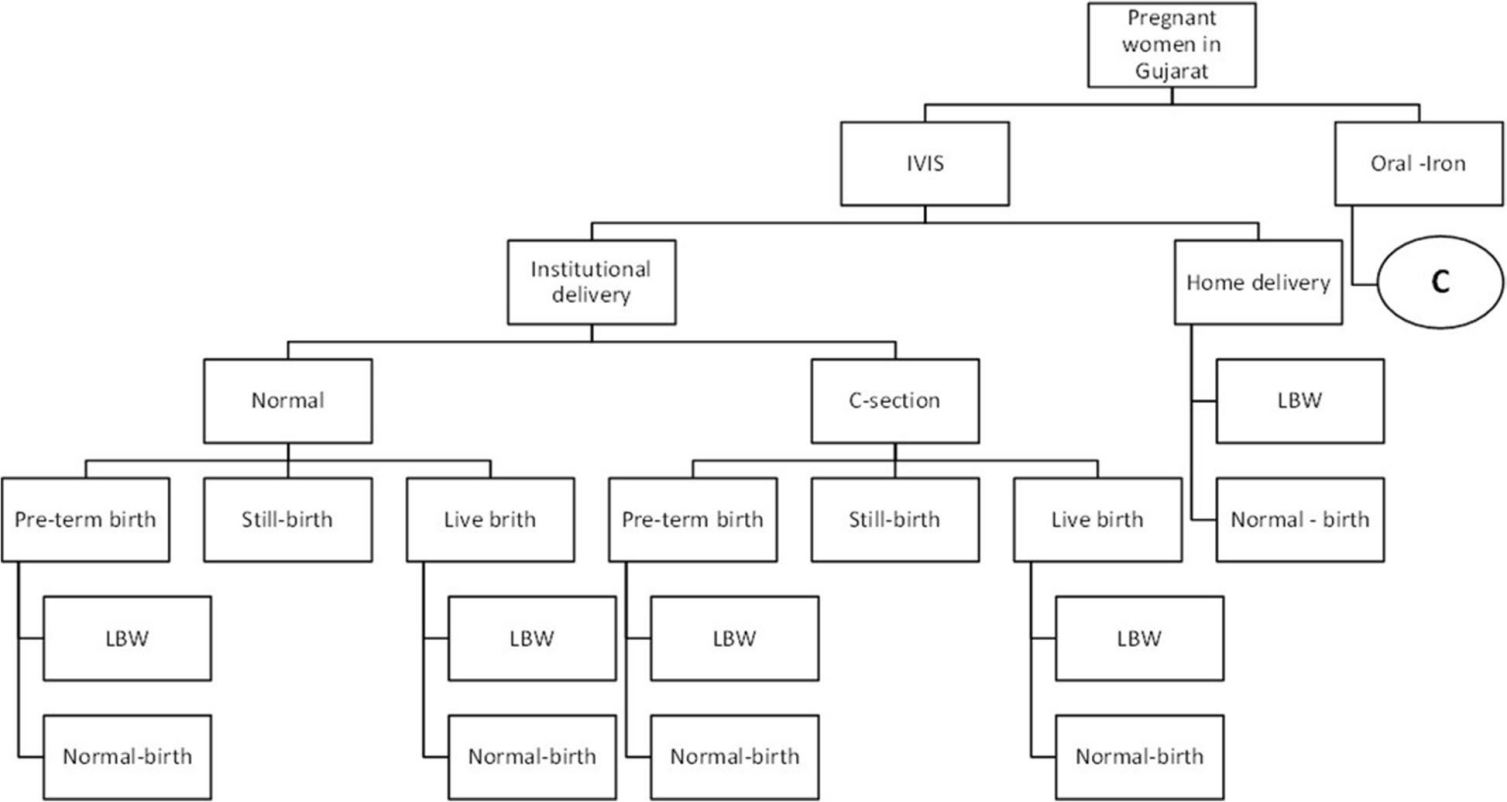
Decision model

MS Excel spreadsheet was used to parameterize a decision tree, which estimated the change in QALYs and cost from a societal perspective (Fig. 1).

Initial outputs in terms of changes in hemoglobin, place of delivery (institutional or home delivery), normal delivery, cesarean section delivery, pre-term birth, still births, live births, low birth-weight, and normal birth weight babies a model was created to estimate the net QALY gained.

To populate the decision tree, primary data was used to derive transition probabilities along with the other data Table 1.

**Table 1:**
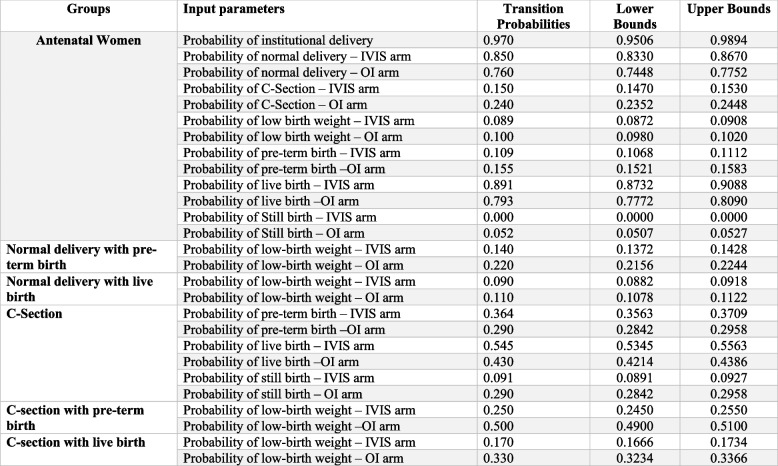
Input parameters used for cost-effectiveness analysis of IVIS programme

The Incremental Cost-Effectiveness Ratio (ICER) calculations combined both costs and outcomes. The study had a time horizon of one year, with a 3% discount applied, and the CEA results were reported as cost per QALY gained. A one-way sensitivity analysis was conducted, where model parameters were varied to assess parameter uncertainty. The ICER values were used to create a tornado chart, which illustrates changes in selected variables and their impact on the results.

## Results

### Study participants

A total of 188 pregnant women were included in the study the IVIS group had 82 and the OI group reported 106 enrollments. Table 2 presents district-wise enrollment in the intervention and control arms. The study included five follow-up examinations. Up to 2nd follow-up, all women were tracked; however, 183 (97.3) women were followed up during the 3rd visit and 170 (90.4) during the 4th visit. The 5th follow-up during the post-partum period witnessed a reduction in the follow-up of pregnant women to nearly half of 142 (75.5). The primary reasons for fewer follow-up visits were migration and services from private providers Table 2.

**Table 2:**
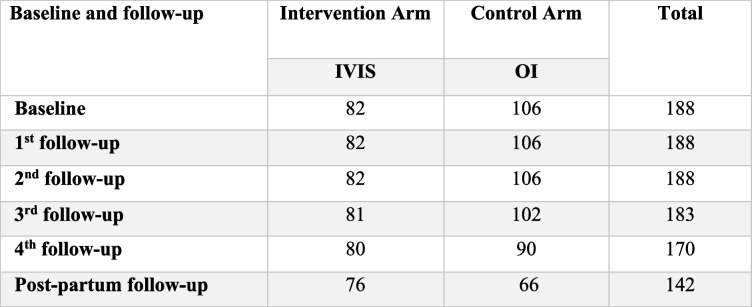
Pregnant women enrolled and follow-up

### Change in mean hemoglobin level

**Fig. 2:**
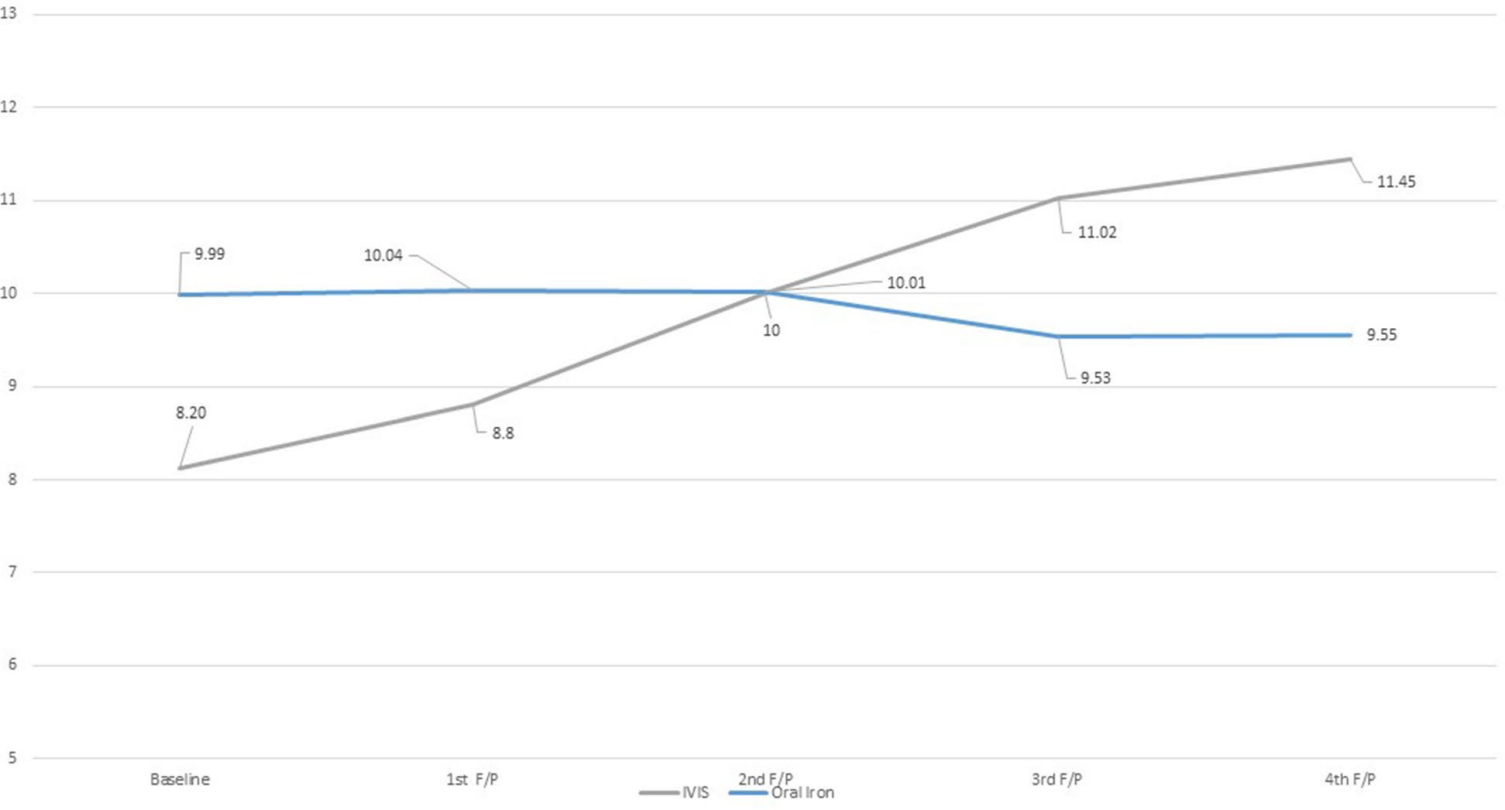
Change in the mean Hb

The study reported a change in mean hemoglobin (Hb) levels across the intervention and control arms. An incremental mean change in Hb was noted in the IVIS arm (11.45 g/d from 8.2 g/dL) at the time of the fourth follow-up, 16 weeks from the baseline (Fig. 2 In the control group, the average Hb level decreased to 9.55 g/dL during the fourth follow-up from the baseline measurement of 9.99 g/dL (Fig. 2).

### Side effects

Table 3 shows more side effects (60%) were reported from the control arm than from the intervention arm. Approximately 60.4% of pregnant women enrolled in OI (*n* = 64/106) reported side effects, while only 10.9% of women in the IVIS (*n* = 9/82) arm reported side effects. In the intervention arm, the side effects were limited to pain at the injection site (*n* = 7) and muscle spasm (*n* = 2) in the IVIS group, and only one patient reported pain at the injection site as a side effect. No major events of adverse drug reaction (ADR) were reported in either arm. Any reported side effects in the intervention group were addressed at the PHC, while 36% of the reported side effects were managed in the OI group. The intervention arm reported 100% compliance in the IVIS and FCM groups within the intervention arm. All participants completed the treatment, whereas 73% compliance was noted in the control arm (OI therapy group). A major reason for discontinuation was side effects, migration, and access to private providers.

**Table 3:**
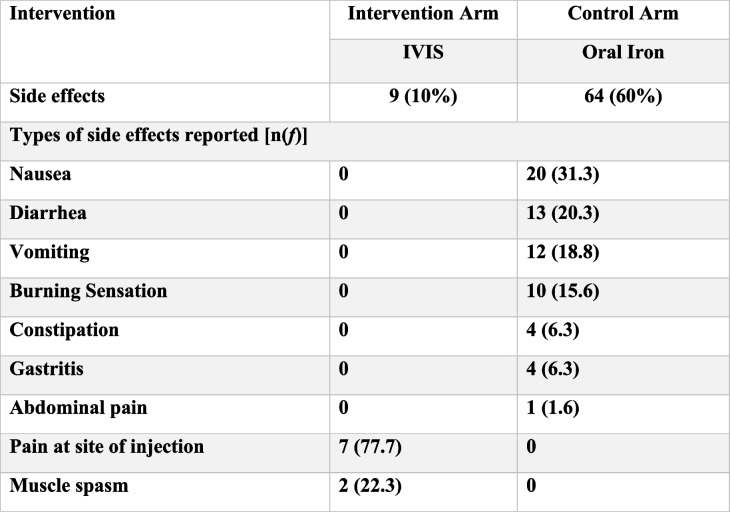
Side effects across interventions (frequency/%)

### Delivery outcomes

We assessed the outcomes of IVIS in the intervention group and OI in the control group and gathered data on delivery outcomes of 76 (out of 82) from the intervention arm and 66 (out of 106) from the control arm. Approximately 97% of participants in the intervention arm had institutional delivery, and the rest (3%) recorded home delivery. Of the total institutional deliveries, 85% were delivered normally, which was higher than that of the control arm and slightly lower incidence of cesarean section delivery (15% and 24%, respectively, in the intervention and control arms). Table 4 presents the details of the key outcomes. We could not gather data on complications such as post-partum hemorrhage (PPH), requirement of blood units during delivery, maternal mortality due to PPH, and early neonatal mortality as all staff were engaged in COVID-19-related duties. Therefore, we restricted our analysis to low and normal birth weights as outcomes and QALY as the model outcomes.

**Table 4:**
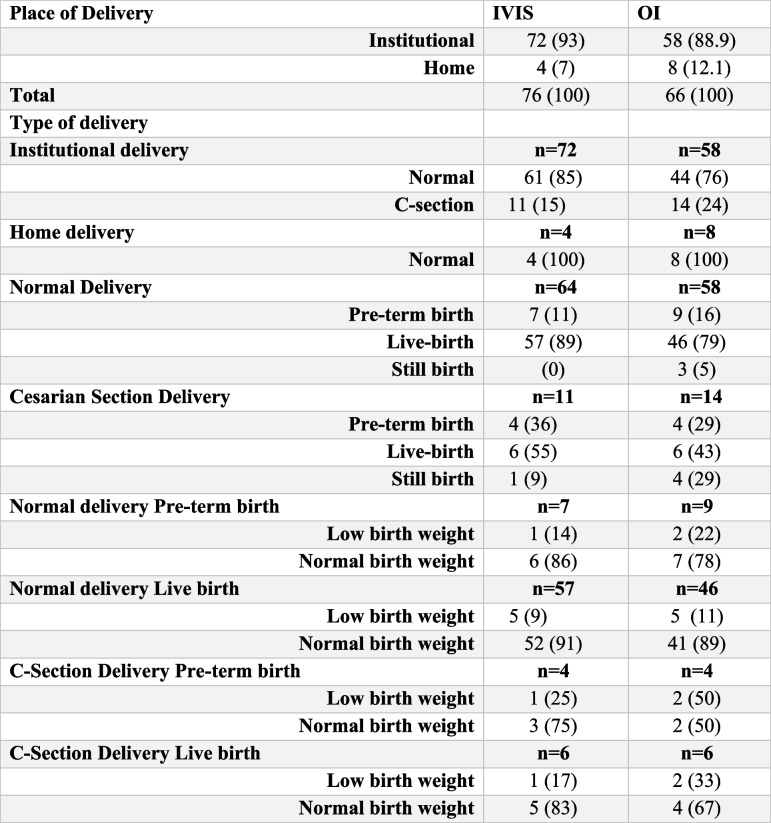
Delivery outcomes

### Health related Quality of Life (HQoL)

The baseline and fourth follow-up (16 weeks following baseline) data show the mean difference in the EQ5D5L score. The mean score was improved in both arms; however, the intervention arm noted more improvements in 5D and 5 L. Table 5 shows EQ5D5L. The EQ5D5L utility index value was significantly lower in the control group as compared to both the intervention arm and baseline values.

**Table 5:**
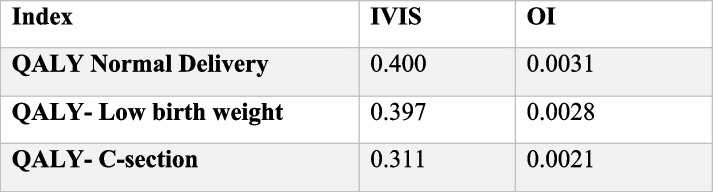
EQ5D5L Utility Index in intervention and control arm

### Costs

The total costs were US$ 7,480 and US$ 5,379 for the IVIS and OI groups, respectively. The discounted cost per beneficiary for the IVIS was US$ 87 and US$ 49 for the OI group (Table 6). The additional cost of complications in delivery for normal delivery and cesarean section was calculated to assess the cost of pre-term birth.

**Table 6:**
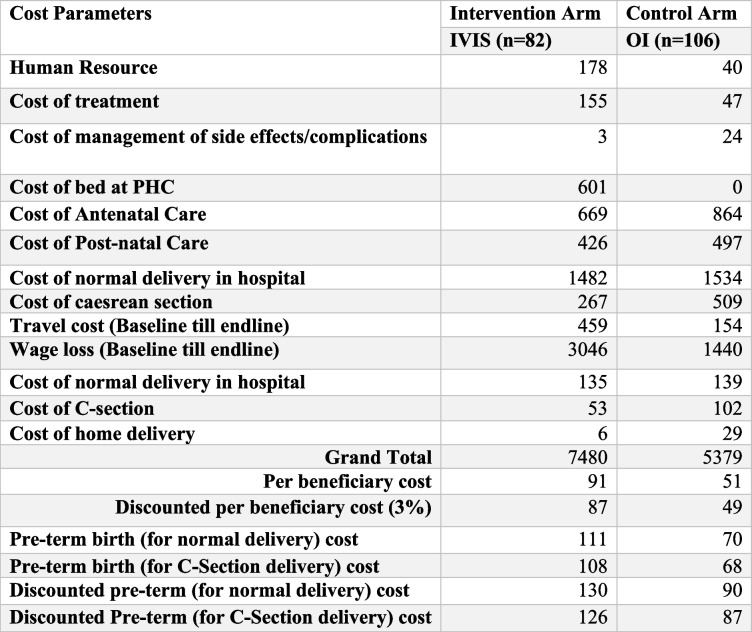
Overall Cost from Societal Perspective for Each Arm

All costs in USD

### Incremental cost and effectiveness

The cost of a decision tree is obtained by adding up the costs associated with each pathway, which is calculated by multiplying the probability of each event by its respective cost. The study also suggests that IVIS incurs an incremental cost of US$9.84 per QALY from a societal perspective, which accounts for approximately 0.49% of India's per capita GDP, as shown in Table 7. Therefore, based on the cost-effectiveness analysis, the IVIS intervention may be considered a cost-effective option.

**Table 7:**
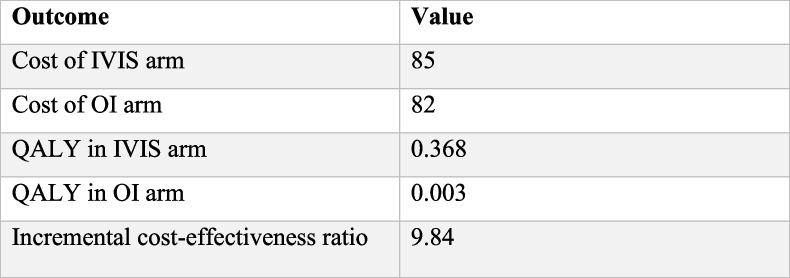
Model outcome summary for IVIS therapy for moderate and severe anemic pregnant women

**Fig. 3:**
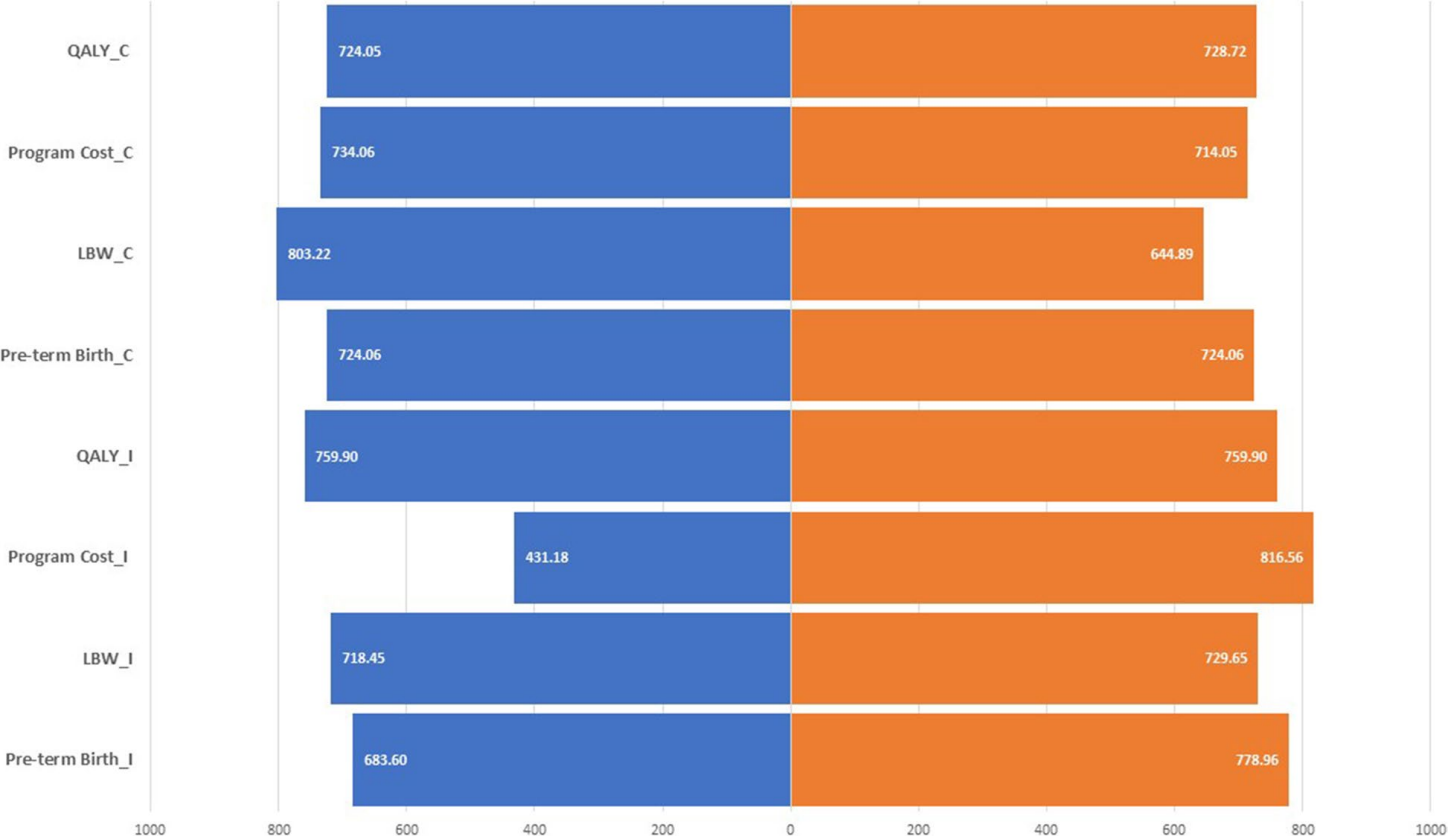
Tornado diagram of cost-effectiveness of IVIS and OI therapy

To conduct a sensitivity analysis, a one-way approach was adopted. The simulations conducted as part of this analysis are illustrated in Fig. 3. The tornado diagram in the one-way sensitivity analysis indicates that the ICER is minimally impacted when certain input parameters are varied. Specifically, the cost of the intervention arm, the incidence of low birth-weight, and pre-term birth reported in the control arm were identified as the key parameters influencing the model (Fig. 3).

## Discussion

We aimed to evaluate the cost-effectiveness of IVIS as compared to oral iron for treating moderate and severe anemia among pregnant women within a real-time setting in Gujarat. The mean increment in the Hb level was higher in the IVIS group than that reported in the Oral Iron group. Thus, IVIS is safe and effective during pregnancy. Compared to OI, IVIS led to a more rapid improvement in anemia. This finding has also been observed in other studies. [9, 16–22].

IVIS is well-suited for treating anemia among pregnant women with lower hemoglobin levels in the second trimester because it increases hemoglobin at a faster rate. According to studies conducted by Neeru, Nair [18], and Rai and Neogi et al. [18] “there was a highly significant difference in hemoglobin levels after treatment between the two groups.” Al et al. also observed that the treatment of pregnant women through IVIS achieved statistically significant higher hemoglobin levels (*P* ≤ 0.001) in a shorter period (*P* ≤ 0.001)” [23]. The present study provided iron supplementation to the IVIS group in order to adhere to government guidelines. A similar approach was reported in Bayoumeu et al.'s study, [18] where iron supplement was continued after IVIS treatment, similar to the study conducted by Neeru, Nair, and Rai (2012), [18] wherein the group treated with IVIS maintained high concentration of hemoglobin with routine supplementation of OI after the treatment.

In India, due to a higher overall prevalence of anemia (66.4%) among pregnant women, as reported in the National Family Health Survey-5, [23] oral supplementation is deemed necessary essential even if iron stores are normal. According to a study conducted by Neeru, Nair, and Rai [16], absorption slowed down once anemia was corrected with OI, unlike that reported in the parenteral iron-treated group. This could be the reason why iron stores are not adequately replenished with OI as effectively as with intravenous iron.

While several studies in India have introduced intravenous route for parenteral iron administration, they reported side effects such as pain and staining at the injection site [24, 25], In the present study, we found the intervention arm with parenteral iron therapy had reported fewer side effects compared to the control arm with oral iron therapy. These side effects may lead to the discontinuation of OI supplementation. Interestingly, we found the Intervention group reported 100% compliance to therapy, whereas in the control group compliance to therapy rate was 73%.

In terms of cost, the Intervention group had a higher cost than the control group. However, it is important to mention that the cost of managing side effects, complications during normal and c-section delivery, and user cost (home delivery) were higher in control group. This means that Intervention certainly reduces user costs significantly and health system costs in the management of complications.

The present study reported an improvement in mean hemoglobin after treatment and in the birth weight of babies. Similar findings have been reported in a study conducted in Northeast India [26]. Previous studies have compared the cost-effectiveness of IVIS with that of Oral Iron therapy and have found that IVIS intervention is promising [27].

Along with clinical effectiveness and compliance, a recent cost-effectiveness study based on a randomized control trial in India also found treatment of anemia among pregnant women through IVIS to be costlier, but also more effective than OI therapy [28]. ICER was calculated per safe delivery as INR 31,951 (US$ 445.2). Our study included pregnant women with moderate and severe anemia and found it to be very cost-effective.

### Limitations

Several limitations in assessing cost-effectiveness are highlighted. Data on some clinical disorders, like complications during pregnancy, maternal mortality and complications due to postpartum hemorrhage (PPH), early neonatal mortality, and the requirement of blood transfusion during delivery could not be collected. Therefore, we focused on pre-term birth, still birth, live birth, low birth-weight babies, and normal weight babies as the health outcomes for our economic modelling. Long-term effect of the treatment on maternal and fetal health were not explored in this study.

## Conclusion

Despite these limitations, the present study holds critical value in evidence generation on IVIS intervention and complements national strategies to support policy decisions for scale-up. The study demonstrated a statistically significant improvement in the mean hemoglobin level in the IVIS group compared to the group with only oral iron supplements. Despite being cost-intensive, we conclude IVIS to be more effective than oral therapy in treating moderate and severe anemia among pregnant women. Moreover, the treatment was well-tolerated, with fewer reported side effects than oral iron supplementation.

## Data Availability

Data are stored in the encrypted and password-protected computer system at the institution. Access to records and study data is restricted to study personnel. Study data is de-identified and stored separately from the data.

## Abbreviations

IVIS: Intravenous Iron Sucrose
OI: Oral Iron
IDA: Iron-Deficiency Anemia
Hb: Haemoglobin
ICER: Incremental Cost-effectiveness Ratio
GDP: Gross Domestic Product
ANC: Antenatal care
CHC: Community Health Centre
GOI: Government of India
HRQoL: Health-related quality of life
I-NIPI: Intensified National Iron Plus Initiative
INR: Indian Rupee
IV: Intravenous
ADR: Adverse Drug Reaction
MoHFW: Ministry of Health and Family Welfare
PHC: Primary Health Center
QALYs: Quality Adjusted Life Years
USD: United State Dollar
VAS: Visual Analog Scale
